# Optical Characterization of Strong UV Luminescence Emitted from the Excitonic Edge of Nickel Oxide Nanotowers

**DOI:** 10.1038/srep15856

**Published:** 2015-10-28

**Authors:** Ching-Hwa Ho, Yi-Ming Kuo, Ching-Hsiang Chan, Yuan-Ron Ma

**Affiliations:** 1 Graduate Institute of Applied Science and Technology, National Taiwan University of Science and Technology, Taipei 106, Taiwan; 2 Graduate Institute of Electro-Optical Engineering and Department of Electronic and Computer Engineering, National Taiwan University of Science and Technology, Taipei 106, Taiwan; 3 Research Center for Applied Sciences, Academia Sinica, Taipei 115, Taiwan; 4 Department of Physics, National Dong Hwa University, Hualien 974, Taiwan

## Abstract

NiO had been claimed to have the potential for application in transparent conducting oxide, electrochromic device for light control, and nonvolatile memory device. However, the detailed study of excitonic transition and light-emission property of NiO has rarely been explored to date. In this work, we demonstrate strong exciton-complex emission of high-quality NiO nanotowers grown by hot-filament metal-oxide vapor deposition with photoluminescence as an evaluation tool. Fine and clear emission features coming from the excitonic edge of the NiO are obviously observed in the photoluminescence spectra. A main excitonic emission of ~3.25 eV at 300 K can be decomposed into free exciton, bound excitons, and donor-acceptor-pair irradiations at lowered temperatures down to 10 K. The band-edge excitonic structure for the NiO nanocrystals has been evaluated and analyzed by transmission and thermoreflectacne measurements herein. All the experimental results demonstrate the cubic NiO thin-film nanotower is an applicable direct-band-gap material appropriate for UV luminescence and transparent-conducting-oxide applications.

The iron Trial group (Fe, Co, Ni) monoxides have received more attentions on various branches of magnetic study owing to the large magnetic pole created by unpaired electrons. However nowadays the optical research of the oxides is not popular and very rare on exploring the optoelectronics property of the iron-Trail oxide has been found owing to the lack of high-quality crystal and the difficulty on performing optical-characterization tool efficiently. NiO had been claimed to be a NaCl-type^1^ antiferromagnetic transition-metal monoxide^2^ with a Néel temperature of about 525 K^3^,^4^. It means a diamagnetic to paramagnetic transition may occur at T > 250 °C for the NiO nanocrystal^5^. The NiO is also a wide-band-gap semiconductor with an energy gap larger than 3 eV. The NiO thin-film nanostructures own some specific electronic and structural properties to lend itself a promising candidate for applications in hydrogen and glucose sensor^6^,^7^, resistive switching memory^8^,^9^, electrochemical electrode for battery and supercapacitor use^10^, electrochromic device^11^, and dye-sensitized and hybrid thin-film solar cells^12^,^13^,^14^,^15^. The behaviors of electrochromic and resistive switching in the NiO can be attributed to the mobile ions (Ni^2+^, OH^−^, etc.) and defects (vacancy, interstitial, or hole) migrated in the nickel oxide under different electric fields for changing the electronic structure or band-gap state. Besides, the formation of a specific nanoarrow or nanotower of NiO can also provide the NiO nanostructure a potential capability for application in light-emission device or field-emission display. Despite the versatile applications, the key parameter for technological usuage of the NiO nanostructure would be the band-edge characteristic including band-gap nature, exciton, valence- and conduction-band property, wherein the Ni 3*d* electron may play a crucial role in the determination of electronic-band structure of NiO. Especially, the optical behavior of excitonic transition should be crucial for a functional nanomaterial for application in light-emission and nanophotonics devices.

In this paper, the fine and clear excitonic emission features of NiO nanostructure have been obviously displayed by photoluminescence (PL) experiment in the temperature range between 10 and 300 K. We present strong and thorough excitonic luminescences (transitions) of the NiO thin-film nanotowers by using PL and thermoreflectance (TR) experiments. Transmission and TR measurements confirm direct semiconductor behavior of the NiO nanotowers. The O 2*p* and Ni 3*d* states of the NiO nanocrystal can be proposed to dominate the formation of band-edge excitons of the NiO^16^. The experimental results of NiO nanostructures show a prominent excitonic emission at ~3.25 eV and a direct gap larger than 3.51 eV, which can act as an UV (or white-light) luminescence material as well as a transparent conducting oxide (TCO) constituent by the iron-Trial transition element Ni.

## Results

### Crystallinity and stacking formula of the NiO nanocrystals

**Figure 1:**
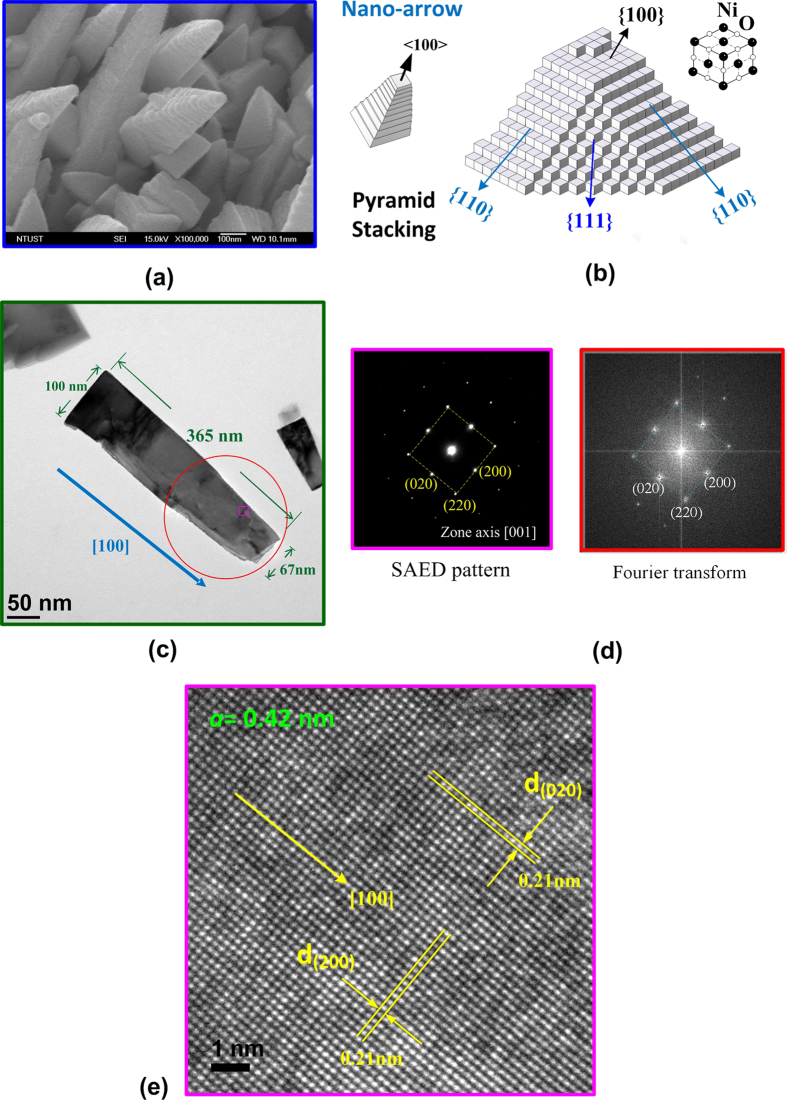
(**a**) FESEM image of the NiO nanotowers (magnification × 10^5^). (**b**) Stacking formula and crystal orientations of the NiO nano-cube particles to form a NiO nanotower. (**c**) TEM image of one single NiO pyramidal nanorod (nanotower) with a length of ~365 nm and a width less than 100 nm. (**d**) SAED and Fourier transform patterns of the NiO nanotower to show well-matched cubic-NaCl structure. (**e**) HRTEM image of the NiO nanocrystal, where the inter-planar spacing of *d*_(200)_ = *d*_(020)_ = 0.21 nm and axial direction of [100] were shown.

Figure 1(a) shows the field-emission-scanning-electron-microscope (FESEM) image of the as-grown NiO thin-film nanostructures with the magnification of ~×10^5^. The shapes of nanostructures reveal nanotower (major), nanocube (minor) and nanorod (minor). The nanotower reveals a stacking formula of pyramid shape with the tip direction along <100> such as a nano-arrow indicated in Fig. 1(b). The central part of Fig. 1(b) demonstrates the representative scheme of the stacking NiO nano-cube particles that lead to the formation of various crystalline planes of {100}, {110}, and {111} in a pyramidal-type nanotower. The right part in Fig. 1(b) shows the rock-salt unit cell of the cubic NiO. For the formation of the NiO nanostructures grown by hot-filament metal-oxide vapor deposition (HFMOVD), the cation source (Ni^+^) comes from the evaporated Ni gas by heating Ni metal (~1200 °C) while the anion reactant (O^−^) may be the residual oxygen in the growth chamber. The production of nano-cube particles occurs immediately after the mixing of the reactants (Ni^+^ and O^−^) inside the chamber. A dissolution-recrystallization mechanism of the nanostructural oxides^17^,^18^ could be used to explain the formation of the NiO nanostructures. Under a certain oxygen ambient, the concentration of Ni gas source may affect the shape of the formed NiO nanostructures. As the Ni concentration gradually increases from low to high, the morphology of the nano oxide may change from nanowire → nanotower → nanorod (or nanocube) with a larger size. Initially, with a lower Ni concentration, the NiO crystallizes in a cubic rock-salt structure with a faster stacking rate along the <100> direction deposition on the sapphire substrate. NiO nanowires were thus formed. At a higher Ni gas concentration, NiO nanotowers were subsequently formed. This situation can be described as the stacking of the cube particles that decreases from the substrate base toward the tip as shown in Fig. 1(b). The growth of nanotower is due to the interruption of lateral growth of nano-cube particle by the presence of defects. The defects hinder the side growth of nano-cube particles into widened nanowires with larger and uniform width (i.e. rectangular nanorods). Such defects would also block a fraction of the base area and shrink the effective nucleation area during the growth to form a nanotower from base toward the tip. When further increasing the Ni concentration, the lateral growth of the NiO enhances and the tip of the nanotower shortens to form larger nanocubes or cubic nanorods. According to these crystallization mechanisms, the dissolution process is usually easy to happen in the edges of the NiO nanorods (nanotowers). Therefore, if the step-like change of the edge in the NiO nanotower goes slowly and smoothly, the nanotower will become a pyramidal-like nanorod. Figure 1(c) shows the transmission-electron-microscope (TEM) image of one single-pyramidal nanorod (nanotower) with the length of ~365 nm (i.e. width ~100 nm at the bottom and ~67 nm at the tip) grown along <100> direction. Both the selected-area-electron-diffraction (SAED) pattern and Fourier transform image of the NiO in Fig. 1(d) also display well-matched cubic (NaCl) crystalline phase and high crystalline quality of the nanostructure (i.e. clear dot patterns). The high-resolution TEM (HRTEM) image of the NiO nanocrystal [derived from a square region in Fig. 1(c)] is also displayed in Fig. 1(e), where the corresponding lattice spacing [d_(200)_ and d_(020)_ ≈ 0.21 nm] and clear atomic view are demonstrated. The axial direction of the pyramidal nanorod (nanotower) is also shown in the HRTEM image. All the results in Fig. 1 confirmed high quality and cubic crystalline phase (*a* = 4.2 Å) of the NiO nanotowers.

### Luminescence properties of NiO nanotowers

**Figure 2:**
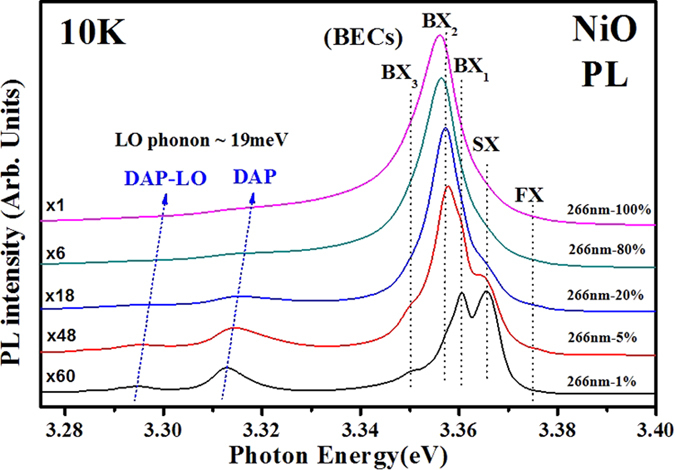
Low-temperature and power-dependent PL spectra of the NiO nanotowers at 10 K. The full power density of the incident 266-nm laser was about 24 W/cm^2^. The excitonic emission of the NiO nanotowers contains FX, BECs, and DAP-related emissions.

Figure 2 shows the low-temperature and power-dependent PL spectra of the NiO nanotowers at 10 K measured by a high-resolution PL spectrometer (iHR 550). The laser power (λ = 266 nm) was varied from full power (100% ~ 24 W/cm^2^) to about 1% using neutral density filter (NDF). Figure S1 demonstrates the experimental representation of the PL measurements from (a) top view and (b) side view of the NiO nanotowers, where the laser spot size is about 2 μm and the averaged thickness of the thin film containing nanotowers is about 1.56 μm. The laser spot size of PL may cover a lot of nanotowers and nanorods with various orientations as shown in Fig. S1. The PL emission of NiO would present an averaged effect of luminescence coming from the cubic NiO nanostructures. As shown in Fig. 2, strong and multiple emissions of the NiO nanotowers are firstly observed by PL measurement. For the low-power spectrum (i.e. 266 nm-1%), three groups of band-edge emissions are found: (1) donor-acceptor pair (DAP = 3.313 eV) and its LO phonon replica (DAP-LO = 3.294 eV), (2) bound-exciton-complexes (BECs) containing peaks of BX_1_ = 3.361, BX_2_ = 3.357, BX_3_ = 3.350, and SX = 3.366 eV, and (3) free-exciton (FX = 3.376 eV) emission of the NiO nanotowers, they are similar to the other cubic oxide nanorods of *c*-In_2_O_3_^19^. The donor-acceptor pair’s distance *r* in columbic energy can be expressed as: E_DAP_ = E_g_ − E_D_ − E_A_ + *e*^2^/(4πε*r*), where E_D_ and E_A_ are the binding energies of donor and acceptor, *e* is the electron charge, and ε is the permittivity of the material. With the increase of the excitation power, the photo-excited DAPs become high density and then the average distance *r* shortened to increase the energies of both DAP and DAP-LO (i.e. parallel movement and separated by a LO phonon energy of ~19 meV shown in Fig. 2). The DAP emission from the donor-acceptor pair’s binding is inferred to come from the intrinsic defects of oxygen and nickel vacancies occurred inside the NiO during the growth process. The appearance of fine band-edge excitonic emissions of DAP, BECs, and FX lends an indication that high crystalline quality of NiO nanostructure is obtained. The result is superior to the other PL measurement result derived from a NiO pellet^20^.

**Figure 3:**
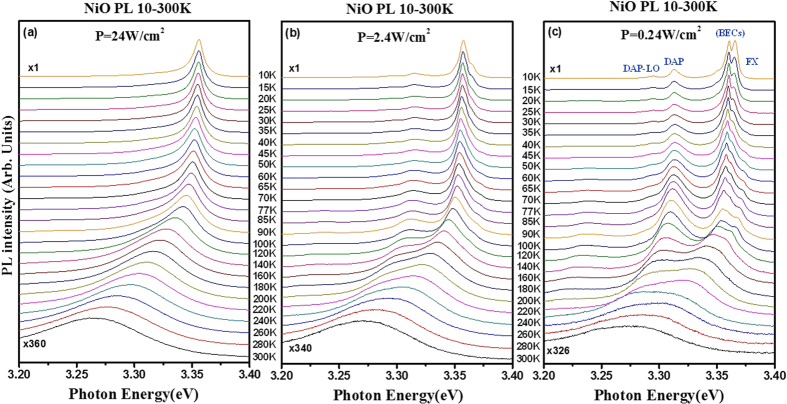
(**a**) Temperature-dependent PL spectra (10–300 K) of the NiO nanotowers obtained at different excitation powers of (**a**) P = 24 W/cm^2^, (**b**) P = 2.4 W/cm^2^, and (**c**) P = 0.24 W/cm^2^, respectively.

As shown in Fig. 2, the BECs are the excitonic emissions bounded by impurities at lower temperatures while the FX peak is not bound but shown lower intensity than those of BECs at 10 K. The SX emission in the BECs is a surface-exciton emission (i.e. trapped by surface states), whose intensity is very sensitive to the laser power (see Fig. 2) and which usually detected in the oxide nanostructures with high surface-to-volume ratio. Figure 3(a,b,c) show the temperature-dependent PL spectra of the NiO nanotowers with different excitation powers of 24, 2.4, and 0.24 W/cm^2^ in the temperature range between 10 and 300 K. Clear and relevant peak features of the PL emissions are detected by the NiO nanotowers from 10 to 300 K with different excitation power densities. For the high-power (P = 24 W/cm^2^) spectra in Fig. 3(a), only one main peak merged from the respective BECs emissions at 10 K (~3.356 eV) is observed. The peak shows energy red shift and line-shape broadened character with the temperature increases from 10 to 300 K. The energy of the peak is about 3.25–3.26 eV at room temperature. This emission peak signifies the characteristic luminescence of the NiO nanostructures. To clarify the origins of the band-edge emissions, the laser power is properly reduced to ~2.4 W/cm^2^ and some of the additional features of DAP, BECs, and FX are approximately appeared and displayed in temperature-dependent PL spectra of Fig. 3(b). For the low-power PL results (P = 0.24 W/cm^2^) in Fig. 3(c), all the band-edge emission features of DAP-LO, DAP, BECs, and FX are obviously detected in the PL spectra from 10 to 300 K. Especially the surface-trapped exciton peak SX decreases vary quickly in comparison with the other bound-excitionic peaks of BECs (BX_1_-BX_3_) following its intrinsic character of temperature-dependent PL intensity change. As the temperature increases from 10 to 300 K, the peak intensity of FX decreases more slowly than the other bound-exciton (BECs) emissions. The FX eVentually dominates the excitonic emission of NiO with T ≥ 140 K as eVident in Fig. 3(c). The DAP related emissions (DAP and DAP-LO) also show a red-shift behavior similar to the other BECs features. The DAP and FX peaks are finally merged together and predominate the main excitonic emission of the NiO nanotowers at room temperature. The temperature dependence of energy of the band-edge emissions FX, BECs, and DAP in Fig. 3(c) has been analyzed and shown in Fig. S2 and the related discussions are described in the supporting information.

## Discussion

### Optical investigation of the band-edge structure of NiO

**Figure 4:**
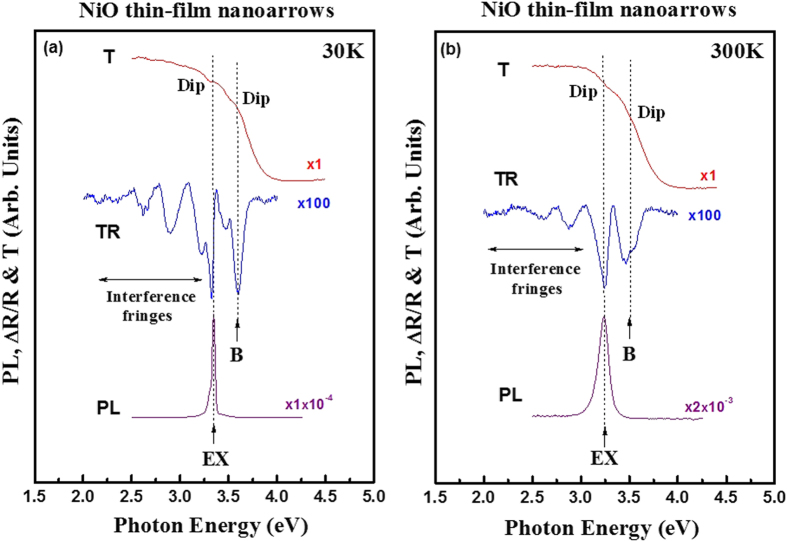
Transmittance (T), thermoreflectance (TR), and photoluminescence (PL) spectra of the NiO thin-film nanostructures at (a) 30 K and (b) 300 K.

To identify the near-band-edge structure of the cubic NiO thin-film nanotowers, transmission, TR, and low-resolution PL measurements are simultaneously carried out. The cubic-NaCl phase of the NiO nanotowers can be verified and identified again using Raman measurement as indicated in the supporting information of Fig. S3. Shown in Fig. 4 are the transmittance (T), TR, and PL spectra of the NiO thin-film nanotowers at (a) 30 K and (b) 300 K, respectively. The optical spot size of incident light of the T and TR measurements is about 0.1 mm^2^. The size is much larger than those of the NiO nanostructures. The experimental results of T and TR will also present an averaged optical effect of the nanocrystals. The transmittance spectrum at 300 K in Fig. 4(b) shows an obvious absorption edge between 3.05 and 3.88 eV, and this edge increases to about 3.15 and 3.98 eV (shift ~100 meV) when the temperature lowered down to 30 K [see Fig. 3(a)] following the general semiconductor behavior. Especially the transmittance spectra at 30 and 300 K simultaneously show two dips that matched well with the two excitonic transitions measured by TR measurements with EX = 3.35 and B = 3.61 eV at 30 K and EX = 3.25 and B = 3.51 eV at 300 K, respectively. The lower-energy feature of EX also agrees well with the energy position of the PL peak as eVident in Fig. 4. This result identifies the main excitonic transition of the NiO nanotower would be coming from the EX exciton. The EX exciton measured in the high-resolution PL measurement of NiO at 10 K can be decomposed into DAP, BECs, and FX, etc. such as the situation shown in Fig. 3(c). The TR measurement can perform periodically thermal modulation of the NiO nanostructures to form easily-resolved derivative spectral-line features of EX and B in Fig. 4^21^,^22^. The TR result also facilitates the detection of complete band-edge transitions of EX and B which complementary to that of the PL with the detection of only one EX feature in Fig. 4. The NiO has been claimed to have the possibility to form several *p*-*d* localized excitons intermediate inside the forbidden gap^16^. The EX and B features in the TR spectra can hence be assigned as the band-edge excitionic transitions from Ni 3*d* (*t*_*2g*_) valence band to those of Ni 3*d*^8*^ − O 2p^*^ exciton states (see the band scheme of Fig. S4). The energy of PL emission peak (EX) agrees well with the band-edge transition of the NiO in Fig. 4. This result also sustains that all the PL signals are coming from the band-edge excitons of the NiO nanostructures. There are some interference fringes found in the TR spectra below the energy position of the EX transition in Fig. 4. They are usually coming from the parallel interfaces of the thin-film oxide in the transparent region (below band edge) with a relationship of *h*·Δν = 1240/(2·*n*·*d*·cos ϕ), where *h*·Δν is the energy difference between two fringes, *n* is the refractive index, *d* is thin-film thickness, and cos ϕ ≈ 1 signifies for a nearly normal-incidence condition of the light. It can be calculated from the TR fringes in Fig. 4 to get the index of refraction of NiO at ~2.8 eV was approximately *n* ≈ 2.1 at 300 K. The value of refractive index approximately matches well with the estimation from dielectric constant of ε_r_ ~ 4.4 of a NiO thin film^23^ calculated by the relation 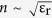. Besides, it should be noted that the PL spectra at 30 and 300 K in Fig. 4 do not show any yellow-green visible luminescence from the NiO nanotowers. The NiO contains high-density vacancies of O and Ni will emit strong yellow-to-green visible luminescences^20^. The absence of the visible luminescence in Fig. 4 reveals good crystalline quality of the as-grown NiO nanotowers. However, for a white-lightening application, a suitable amount of Ni and O vacancies can be properly designed to introduce into the NiO nanostructures for rendering some applicable visible emissions.

In summary, high-quality cubic NiO nanotowers have been successfully grown by the HFMOVD method on sapphire with the axial direction grown along <100>. Strong UV excitonic emission containing DAP, FX, and BECs are firstly observed in the NiO nanotowers by PL. The result indicates good crystalline quality of the nanostructures. An excitonic transition of the NiO nanoarrows at ~3.25 eV (EX) can be simultaneously detected by transmittance, TR, and PL measurements at 300 K. The EX transition dominates the main band-edge emission of the NiO. One additional feature of exciton B at ~3.51 eV can also be detected by the TR and transmission measurements. The existence of EX and B excitons verifies the direct band-gap nature of the NiO nanocrystal, and the transitions are originated from some local excitonic states constituted by Ni 3*d*^8*^ − O 2p^*^ in the band gap of nickel oxide. Based on the experimental analysis, the band edge structure of the NiO nanocrystrals is thus realized. The experimental result demonstrates a potential function of NiO for application in white-light luminescence device.

## Methods

### Preparation of NiO nanostructures

Large-scale and high-density NiO nanotowers were grown by HFMOVD on sapphire substrate^24^. Pure nickel wire (99.9%) with 1-mm diameter was heated to ~1200 °C in an evacuated chamber with a pressure of ~2 Torr. The heating process maintained for 50 min to generate gaseous Ni. The evaporated Ni reacted with residual oxygen (or leakage air) to form NiO vapor and then was condensed onto a pre-cleaned sapphire {100} substrate to form the one-dimensional stacking NiO nanotowers. X-ray diffraction measurements confirmed cubic crystalline phase of the as-grown NiO nanostructures.

### Optical measurements

The PL experiments were implemented by using two different spectrometers with dissimilar resolution^19^. The high-resolution PL measurement was carried out in an iHR550 imaging spectrometer equipped with a 2400 grooves/mm grating acted as the dispersion unit. The low-resolution PL spectra were measured by a QE65000 spectrometer. The silicon CCD array detection was employed in the PL measurements. The pumping light source was a Q-switched 266-nm solid state laser with a spot size reduced to ~2 μm. A set of NDF controlled and changed the incident laser power of the samples. A closed-cycle cryogenic refrigerator (equipped with temperature controller) facilitated the low-temperature and temperature-dependent measurements. The TR experiments were carried out using indirect heating manner with a gold-evaporated quartz plate as the heating element. The NiO thin-film sample was closely attached on the heating element by silicone grease. Periodically on-off heating disturbance of the sample was carried out with applying the AC current to the heating element in a frequency of ~4 Hz. The experimental details were described elsewhere^25^.

## Additional Information

**How to cite this article**: Ho, C.-H. *et al.* Optical Characterization of Strong UV Luminescence Emitted from the Excitonic Edge of Nickel Oxide Nanotowers. *Sci. Rep.* **5**, 15856; doi: 10.1038/srep15856 (2015).

## Supplementary Material

Supplementary Information
