# Supplementary material for: Optical Characterization of Strong UV Luminescence Emitted from the Excitonic Edge of Nickel Oxide Nanotowers
Source: Sci Rep. 2015 Oct 28;5:15856. doi: 10.1038/srep15856 (PMC4623674; doi:10.1038/srep15856)
Supplement: Supplementary Information [file srep15856-s1.doc]

Supporting Information

Optical Characterization of Strong UV Luminescence Emitted from the Excitonic Edge of Nickel Oxide Nanotowers

# Ching-Hwa Ho,^1,2,*^ Yi-Ming Kuo,^1^ Ching-Hsiang Chan,^2^*,*^3^ and Yuan-Ron Ma^4^

^1^ *Graduate Institute of Applied Science and Technology, National Taiwan University of Science and Technology, Taipei 106, Taiwan*

^2^ *Graduate Institute of Electro-Optical Engineering and Department of Electronic and Computer Engineering, National Taiwan University of Science and Technology, Taipei 106, Taiwan*

^3^ *Research Center for Applied Sciences, Academia Sinica, Taipei 115, Taiwan*

^4^ *Department of Physics, National Dong Hwa University, Hualien 974, Taiwan*

^*^Corresponding author; E-mail: [chho@mail.ntust.edu.tw](mailto:chho@mail.ntust.edu.tw)

Figure S1. (a) Top view and (b) cross-section view of the NiO thin-film nanotowers available for showing the measurement indication of the PL experiments. The laser spot size impinged on the NiO nanostructures was about 2 μm [see the indication in (a)] and the optically excited luminescence from the NiO was collected and focused into a spectrometer as the representation shown in (b). The thin-film nanostructures grown on sapphire can be regarded as initially a compact and continuous film is deposited onto the substrate and then the nanotowers (nanorods) are stacking on the condensed film. The averaged thickness of the thin film (containing nanotowers) is about 1.56 μm.

Figure S2. Temperature dependence of transition energies of the band-edge excitonic emissions FX, SX, BX_1_, DAP, and DAP-LO that obtained by PL measurements in Fig. 3(c). The hollow symbols are the experimental data and solid lines are the least-square fits to an expression containing Bose-Einstein occupation factor for phonons:^26^ , where *E* (0) is the energy at T=0 K, *a_B_* represents the strength of electron (exciton) and average phonon interaction, and $\hbar\omega_{B}$ corresponds to the average phonon energy.

Figure S2 shows the dependence of energy vs. temperature for the PL peaks observed in the low-power PL spectra of the excitonic-edge emissions in Fig. 3(c). The Bose-Einstein analysis of the temperature-energy shift of the FX, SX, BX_1_, DAP, and DAP-LO emissions revealed that the parameters E(0), *a_B_*, and $\hbar\omega_{B}$ can be obtained from the least-square fits (as solid lines) shown in Fig. S2 and the values are listed in Table S1 for comparison. The higher value of *a_B_* of the free-exciton emission FX indicates that the temperature-energy shift of the FX transition is faster than those of the other defect (or impurity) related bound excitons SX, BX_1_, DAP, and DAP-LO. This phenomenon is similar to the other oxide semiconductor such as ZnO.^27^ The average phonon energies of $\hbar\omega_{B}$ obtained in Table S1 are comparable for all the excitonic emissions occurred inside the same NiO nanotowers.

Table S1. Values of fitting parameters of Bose-Einstein type analysis which describe temperature dependences of transition energies of FX, SX, BX_1_, DAP and DAP-LO emissions.

| Feature | E(0)  (eV) | *a*_B_  (meV) | (meV) |
| --- | --- | --- | --- |
| FX | 3.375±0.001 | 67±10 | 23±2 |
| SX | 3.363±0.001 | 30±5 | 22±2 |
| BX_1_ | 3.360±0.001 | 29±5 | 23±2 |
| DAP | 3.313±0.001 | 31±4 | 24±2 |
| DAP-LO | 3.294±0.001 | 31±4 | 24±2 |

Figure S3. Room-temperature Raman spectrum and the assignments of lattice vibration modes of the as-grown NiO thin-film nanotowers.

Figure S3 shows the Raman spectrum of the NiO nanotowers in the energy range between 400 and 1800 cm^-1^ and the assignments of the Raman modes are also included. At first, the first-order transverse optical phonon [1P(TO)] was observed at about 420 cm^-1^ and the first-order longitudinal optical phonon [1P(LO)] was at ~550 cm^-1^, similar to the other reported NiO thin film.^28^ The intense and sharpness of the 1P(TO) peak indicates the crystal size reduction of the formed NiO (i.e. nanostructures).^29^ The smaller intensity of the 1P(LO) peak (i.e. as shoulder like in Fig. S3) also reveals good quality of the nanotowers owing to the degradation of the disorder-induced 1P band at ~550 cm^-1^. The 740 cm^-1^ peak was a two-TO-phonons band, 906 cm^-1^ mode contained TO+LO phonons and the 1100 cm^-1^ mode [2P(2LO)] consisted of two LO phonons. The 1500 cm^-1^ peak is closely related to 2 magnetrons (2M) scattering which may disappear when the temperature of the NiO higher above Néel temperature with a diamagnetic to paramagnetic transition.^30^ All the Raman vibration modes shown in Fig. S3 verified that the as-grown NiO nanotowers belong to a cubic rock-NaCl structure.

Figure S4. Representative band-edge scheme of the NiO nanotowers (with energy scale) determined by the optical measurements of TR and PL experiments.

Figure S4 depicts the representative scheme of the band-edge structure of NiO nanotowers referred to the experimental results of PL, TR and transmittance measurements. For the valence band, the major portion is consisted of Ni 3*d* mixed with O 2*p* electrons.^31^ The top of valence band was dominated by Ni 3*d*^8^ (*t_2g_*). The Ni 3*d*^8^ (*t_2g_*) state combined with a lower O 2*p* state constructed the main valence band. The NiO is associated with the presence of so-called “controlled Ni vacancy” (acceptor) to adjust the p-type conductivity behavior of the nickel oxide which located close to the valence-band top during the growth process of the NiO nanocrystals.^5^ For the conduction band edge, there are a lot of Ni 3*d*^8*^-O 2*p*^*^ localized excitons may exist near the conduction-band minimum.^16,32^ The main conduction band can be composed by higher Ni 4*s* and lower Ni 3*d*^8*^ (*e_g_*^*^) antibonding state. The excitonic transitions of EX and B (measured by the TR and transmittance experiments) in Fig. 4 are thus assigned as the direct transitions from Ni 3*d*^8^ (*t_2g_*) to the Ni 3*d*^8*^-O 2*p*^*^ localized excitons as the indication shown in Fig. S4. The strong UV luminescence at ~3.25 eV (i.e. the EX in Fig. 4 by PL) may come from the emission of lowest Ni 3*d*^8*^-O 2*p*^*^ exciton state to the Ni 3*d*^8^ valence band.

Supplementary Information References

26. Viña, L., Logothetidis S., Cardona, M. Temperature Dependence of The Dielectric Function of Germanium. *Phys. Rev. B* **30**, 1979-1991 (1984).

27. Zhang, X. *et al.* Synthesis of Large-Scale Periodic ZnO Nanorod Arrays and Its Blue-Shift of UV Luminescence*. J. Mater. Chem.* **19**, 962-969 (2009).

28. Jiang, D. Y., Qin, J. M., Wang, X., Gao, S., Liang Q. C., Zhao, J. X. Optical Properties of NiO Thin Films Fabricated by Electron Beam Evaporation. *Vacuum* **86**, 1083-1086 (2012).

29. Mironova-Ulmane, N. *et al.* Raman Scattering in Nanosized Nickel Oxide NiO. *J. Phys.: Conf. Ser.* **93**, 012039 (2007).

30. Dietz, R. E., Parisot, G. I., Meixner, A. E. Infrared Absorption and Raman Scattering by Two-Magnon Processes in NiO. *Phys. Rev. B* **4**, 2302-2310 (1971).

31. Anisimov, V. I., Solovyev I. V., Korotin, M. A. Density Functional theory and NiO photoemission spectra. *Phys. Rev. B* **48**, 16929-16934 (1993).

32. Glosser R., Walker, W. C. Electroreflectance Observation of Localized and Itinerant Electron States in NiO. *Solid State Commun.* **9**, 1599-1602 (1971).
